# Design and psychometric evaluation of the fathers’ fear of childbirth scale: a mixed method study

**DOI:** 10.1186/s12884-021-03696-7

**Published:** 2021-03-20

**Authors:** Seyedeh Fatemeh Ghaffari, Seyed-Hamid Sharifnia, Forouzan Elyasi, Zohreh Shahhosseini, Zohre Mohammadpoorsaravimozafar

**Affiliations:** 1 Mazandaran University of Medical Sciences, Sari, Iran; 2 Amol Faculty of Nursing, Mazandaran University of Medical Sciences, Sari, Iran; 3 Psychiatry and Behavioral Sciences Research Center, Sexual and Reproductive Health Research Center, Addiction Institute, Mazandaran University of Medical Sciences, Sari, Iran; 4 Sexual and Reproductive Health Research Center, Mazandaran University of Medical Sciences, Sari, Iran

**Keywords:** Childbirth, Fathers, Fear, Tocophobia, Psychometric

## Abstract

**Background:**

Fear of childbirth is reported in 13% of fathers, and it may have adverse consequences for the fathers’ health as well as their families. To reduce the fear of childbirth in the expectant fathers, an appropriate screening tool is needed. Due to the lack of a valid and reliable questionnaire to measure fathers’ fear of childbirth, this study was conducted to develop the Fathers’ Fear of Childbirth Scale and evaluate its psychometric properties.

**Methods:**

This mixed method study was conducted in two phases. In the qualitative phase (or item generation), semi-structured interviews were conducted with 20 expectant fathers, and a literature review was performed to generate the Fathers’ Fear of Childbirth Scale items pool. In the quantitative phase (or psychometric evaluation), reliability as well as face, content, and construct validity of this scale were evaluated. To establish construct validity, exploratory and confirmatory factor analyses were performed. Reliability was evaluated through internal consistency and composite reliability measures.

**Results:**

The primary version of Fathers’ Fear of Childbirth Scale contained 32 items, which were reduced to 17 items while establishing construct validity. Exploratory factor analysis extracted two factors, namely fear of childbirth process (12 items) and fear of hospital (5 items). These factors explained 50.82% of the total variance. Goodness of fit indices within the confirmatory factor analysis was acceptable. Internal consistency and composite reliability indices of all the factors were greater than 0.70.

**Conclusion:**

The Fathers’ Fear of Childbirth Scale has a suitable validity and reliability for assessing fear of childbirth in fathers. It is a simple report instrument that can be easily implemented by health care professionals.

**Supplementary Information:**

The online version contains supplementary material available at 10.1186/s12884-021-03696-7.

## Background

Pregnancy, childbirth, and parenting instigate different emotional responses in fathers, including fear of childbirth [1–3]. The fear of childbirth is defined as a negative perception starting in the antenatal period and mostly experienced during labor and delivery [4]. Some biopsychosocial factors may lead to the fear of childbirth in fathers including the interventions during labor and their side effects [5], damage to pregnant mother and her child [5, 6], painful labor and delivery [6, 7], inability to support the spouse [7], disrespectful behaviors by hospital staff [5, 8], and financial constraints [6].

In clinical practice, fear levels are commonly divided into low, moderate, severe, and phobic fear [9]. Some degree of fear of childbirth can be considered normal, but its exacerbation during pregnancy and childbirth is undesirable [10, 11]. According to the existing literature, severe fear of childbirth has been reported in 13% of fathers and it may have poor consequences for the health of fathers as well as their families [8, 12]. Some of those including cesarean delivery [13], mental and physical problems in the expectant fathers [6, 14], poor support from the pregnant mother [5], impairment of father-pregnant mother/child relationship [15], and the unpleasant experience of childbirth [8]. Preparation for childbirth may help distressed expecting fathers to enjoy a more positive childbirth experience [15]. Therefore, fathers at risk of fear of childbirth must be identified and promptly supported by the appropriate services. Screening the expectant fathers experiencing fear of childbirth requires a valid instrument.

To the best of our knowledge, few studies have investigated fear of childbirth in fathers and only a limited number of validated instruments exist to identify this problem [8, 12]. The 52-item questionnaire designed by Ringler [16] and the 33-item Wijma Delivery Expectancy/Experience Questionnaire (W-DEQ) are two instruments administrated to identify fathers with fear of childbirth [15]. Although the original version of W-DEQ was designed to measure the fear of childbirth in women [17], Bergstrom et al. used this questionnaire for expectant fathers by excluding some items irrelevant for men such as some items about labor and delivery processes [18]. Some studies have used the Fear of Birth Scale (FOBS) to measure fear of childbirth in fathers [12, 19, 20]. This scale was also initially introduced to measure the fear of childbirth in women, and there is no evidences about the validity and reliability of this scale for assessment of fathers’ fear of childbirth [12].

It is stated that the content of any tool should be culturally appropriate for the target group for which they are intended [21]. As a valid and reliable tool is needed to investigate fathers’ fear of childbirth, this study aimed to design and examine psychometric properties of the Fathers’ Fear of Childbirth Scale (FFCS) In Iranian setting.

## Methods

### Design and setting

This methodological research was conducted in two phases between May 2019 and January 2020. A qualitative content analysis and item generation were performed in the first phase. The second phase involved a psychometric evaluation of the tool and the assessment of its validity and reliability.

This project was conducted in prenatal clinics of health care centers affiliated to Mazandaran University of Medical Sciences (MAZUMS), Sari, Iran. Sari, the capital of Mazandaran province- northern Iran, is the largest and most populous city in this area. In this city, public coverage includes primary health care. Expectant fathers, who attended in the prenatal visits with their spouses in second or third trimester of pregnancy, were employed.

### Qualitative phase (item generation)

At first for designing the FFCS, semi-structured interviews with the expectant fathers was conducted. During interviews, fathers were held to explore their experiences related to fear of childbirth. Participants selected using purposeful sampling with maximum variation in terms of age, education, and occupation. The interviews lasted 60–90 min and were recorded by the researcher. All interviews were held in Persian and by the same interviewer (A Master of Science in Midwifery on the supervision of research team including a psychiatrist and a reproductive health specialist). Data saturation was reached after 20 interviews. After transcribing the interviews, they were analyzed through the conventional four-step content analysis approach. Accordingly, each interview was divided into meaningful units, which were condensed and coded. Then, the resulting codes were grouped into categories and subcategories [22]. Data was managed using the MAXQDA 10 software, and trustworthiness was ensured via Guba and Lincoln’s criteria, including credibility, dependability, confirmability, and transferability [23].

Also, in this phase, a comprehensive search in the relevant databases such as: Scopus, Science Direct, Psych Info, PubMed, and Cochrane were conducted. Keywords and syntaxes were as follows: [“Fear” OR “Tocophobia”] AND [“Childbirth” OR “Delivery” OR “Parturition” OR “Birth”] AND [“Pregnancy” OR “Gestation”] AND [“Father” OR “Men” OR “Couple” OR “Paternal”] AND [“Related Factors” OR “Influence Factors” OR “Contributed Factors”] AND [“Psychometry” OR “Psychometric”] AND [“Scale” OR “Questionnaire” OR “Tool” OR “instrument”]. The literature review was carried out until saturation was reached for the items of the FFCS. Finally, based on the results of the interviews and literature review, an item pool was generated.

### Quantitative phase (psychometric evaluation)

In the quantitative phase, reliability as well as face, content, and construct validity of the FFCS were established.

#### Face validity

The face validity of the FFCS was evaluated both qualitatively and quantitatively. For qualitative face validity, 10 expectant fathers were invited to comment on the difficulty, appropriateness, clarity, and essentiality of the items. The items were then modified based on their comments. For quantitative face validity, 20 expectant fathers were asked to rate the importance of each item on a 5-point scale ranging from 1 (Not important) to 5 (Very important). The impact score of each item was calculated by multiplying its importance score by the number of fathers who had rated it 4 or 5. An impact score greater than 1.5 was considered appropriate [24].

#### Content validity

Content validity was also evaluated using both qualitative and quantitative methods. For qualitative content validity, 11 experts (i.e., two gynecologists, six reproductive health and midwifery specialists, and three psychiatrists) who were experienced in instrument development were asked to comment on the structure, wording, item allocation, and scoring of the FFCS items. The scale was amended based on their comments. Next, content validity ratio (CVR) and content validity index (CVI) of the primary version of the scale were evaluated. Accordingly, for CVR evaluation the expert group was asked to rate the essentiality of each FFCS item as being “Essential” (score of 1), “Useful but not essential” (score of 2), or “Not essential” (score of 3). Based on the Lawshe Table, items with CVR values less than 0.59 were excluded [25]. For CVI calculation, the same expert group was invited to rate the relevance of each item. Item CVI (I-CVI) with values more than 0.79 were considered appropriate, between 0.79 and 0.70 were revised, and scores below 0.70 were considered unacceptable [24]. In addition, an average scale-level CVI (S-CVI/Ave) was evaluated. An S-CVI/Ave of greater than 0.80 was considered acceptable.

#### Item analysis

Prior to construct validity evaluation, 30 expectant fathers were asked to complete the FFCS. Their responses were used for internal consistency evaluation. Items with an inter-item correlation coefficient of less than 0.30 and greater than 0.80 were omitted.

#### Construct validity

Comrey and Lee (1992) offered a rough rating scale for adequate sample sizes in factor analysis as follows: 100 = poor, 200 = fair, 300 = good, 500 = very good, 1000 or more = excellent [26]. Therefore, 433 eligible fathers were recruited to complete the 23-items FFCS and socio-demographic checklist for exploratory (200 fathers) and confirmatory factor analyses (233 fathers). The sociodemographic checklist included items on age, level of education, occupation, number of children, and having a wanted pregnancy. Convenience sampling was used to choose the participants from the health care centers affiliated to MAZUMS. Inclusion criteria were basic literacy, no history of hospitalization in psychiatric hospitals, and consent to participate in the study. High-risk pregnancies, any history of a child with physical or mental abnormalities in the family, and chronic maternal illnesses that endanger the mother’s life were exclusion criteria.

For exploratory factor analysis (EFA), sampling adequacy was assessed via the Kaiser-Meyer-Olkin and Bartlett tests. Then, the latent factors of the FFCS were extracted via the maximum-likelihood EFA with Promax rotation. The number of extractable factors was determined via parallel analysis. The minimum acceptable factor loading for the presence of an item in a factor was 0.3, which was calculated using the equation below:

*CV*=5.152÷√ (n-2). Based on the three-indicator rule, each factor had to have at least three items [27]. Items with communality values less than 0.2 were excluded [28].

Using confirmatory factor analysis (CFA), the extracted factor model was evaluated via maximum likelihood estimation by using the following model fit indices: incremental fit index (IFI), comparative fit index (CFI), adjusted goodness of fit index (AGFI), parsimony normed fit index (PNFI), parsimony comparative fit index (PCFI), root mean score error of approximation (RMSEA), and minimum discrepancy function divided by degrees of freedom (CMIN/DF).

#### Normal distribution, outliers, and missing data

Univariate normality was evaluated using skewness (±3) and kurtosis (±8). Multivariate outliers were assessed via the Mahalanobis D squared test (*P* < 0.001). Moreover, multivariate normality was assessed via Mardia coefficient of multivariate kurtosis (< 20) [23]. Missing data was assessed via multiple imputations, and it was replaced with the mean of participants’ scores.

#### Reliability

Cronbach’s alpha, McDonald’s omega, and AIC were calculated to evaluate internal consistency [29]. An acceptable internal consistency involved a coefficient greater than 0.70 and an AIC between 0.20 and 0.40 [27]. The data was analyzed using SPSS-AMOS24 and SPSS R-menu_2.0_.

## Results

### Item generation

Analysis of the interviews resulted in the development of seven main categories that may lead to fear of childbirth in fathers. These categories included harm to the mother’s health, lack of adequate care from the mother, harm to the child’s health, interpersonal and relational factors, expenses, childbirth complications, lack of information about childbirth and its stages, and parenting role.

The review of literature resulted in six categories in terms of factors may lead to fear of childbirth in the expectant fathers including maternal factors (e.g., health and safety of the mother, mother’s capability in childbirth, mother’s pain, mother’s fear, and maternal birth control), paternal factors (e.g., lack of information about childbirth, inability to support the spouse, and to be a good father), child-related factors (e.g., health and safety of the child, infant anomaly, childbirth injuries, and hospitalization in neonatal intensive care unit), health care providers factors (e.g., professional’s competence and behavior), birth process factors (e.g., poor outcome of delivery and assisted delivery), and hospital-related factors (e.g., facilities and equipment).

**Fig. 1:**
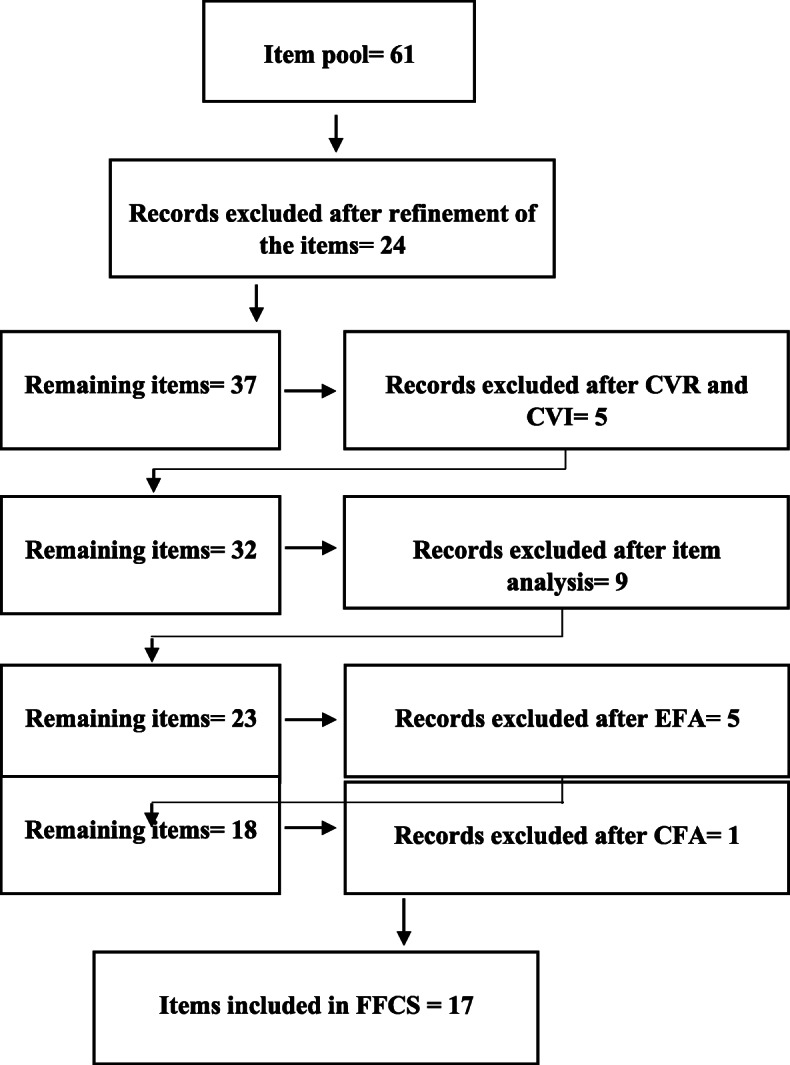
Items selection process

Based on the results of the conducted interviews and the literature review, 61 items were generated. A further refinement of the items reduced the items number to 37 (Fig. 1). These 37 items were grouped into the following categories: fear of maternal-related factors (five items), fear of paternal-related factors (15 items), fear of child-related factors (three items), fear of interpersonal and relational related factors (two items), fear of treatment staff-related factors (four items), fear of birth process-related factors (six items), and fear of hospital-related factors (two items).

### Face and content validity

Three items were revised in the qualitative face validation stage and four items were revised in the quantitative face validation stage due to impact scores less than 1.5. As shown in Fig. 1 content validity resulted in the exclusion of five items due to CVR values less than 0.59. Based on CVI values, no item was deleted and the S-CVA/Ave of the FFCS with 32 remaining items was 0.85.

### Item analysis

Based on the internal consistency evaluation, nine items with inter-item correlation coefficients less than 0.30 were excluded (Fig. 1).

### Construct validity

In total, 433 fathers completed the FFCS with 23 items for factor analysis. Their mean age was 30.22 ± 2.74 years, 60.53% of the fathers had a university degree, 98.3% were employed, 42.81% had no children, and 38.90% had one child. In addition, 86% of the pregnancies were wanted.

A maximum likelihood EFA with Promax rotation was performed on the data obtained from 200 fathers. The Keiser-Meyer-Olkin test value was 0.91 and the Bartlett’s test value was 2985.98 (*P* < 0.001). In this step, five items as a result of communality values less than 0.2 were deleted. A parallel analysis resulted in the extraction of two main factors: fear of childbirth process (12 items) and fear of hospital (6 items). The eigenvalues of these two factors were respectively 5.21 and 3.93, and they explained 50.82% of the total variance of the FFCS (Table 1).

**Table 1:** Factors extracted from FFCS

Factors	Items	Factor loading	*h*^*2*^	*ʎ*	Variance
Fear of Childbirth Process	25. I am afraid that my spouse’s health will be endangered due to childbirth.	0.868	0.822	5.213	%28.96
	9. During my spouse’s childbirth, I will feel fear.	0.751	0.643		
	6. I am afraid that dangerous medical interventions will be needed during childbirth.	0.748	0.561		
	4. As the time of childbirth approaches, my worries increase.	0.736	0.419		
	22. I’m afraid that my spouse’s childbirth will be risky.	0.706	0.609		
	8. During my spouse’s childbirth, I will feel helpless.	0.685	0.333		
	10. During my spouse’s childbirth, I will feel restless.	0.673	0.512		
	26. I am afraid that my child’s health will be endangered due to childbirth.	0.591	0.734		
	3. I worry about the quality of sex with my spouse after childbirth.	0.533	0.272		
	24. I will feel fear because of my spouse’s pain.	0.523	0.660		
	15. Because of my spouse’s fear of childbirth, I feel fear.	0.482	0.525		
	17. I am afraid that I am not capable enough to support my spouse during childbirth.	0.489	0.595		
Fear of Hospital	31. I am afraid that the hospital staff will not have enough skills to perform a safe childbirth.	0.997	0.883	3.936	%21.86
	32. I am afraid that the hospital will not have enough facilities and equipment for a safe childbirth.	0.947	0.784		
	28. I am afraid that the hospital staff will not take enough care of my spouse.	0.843	0.858		
	29. I’m afraid the hospital staff won’t treat me and my spouse respectfully.	0.762	0.879		
	30. I am afraid that my child will be hospitalized in the neonatal intensive care unit after birth.	0.622	0.672		
	27. It will be difficult for me to pay for the hospital.	0.609	0.289		

*Abbreviations*: *ʎ* Eigenvalue, *h*^*2*^ communality

**Fig. 2:**
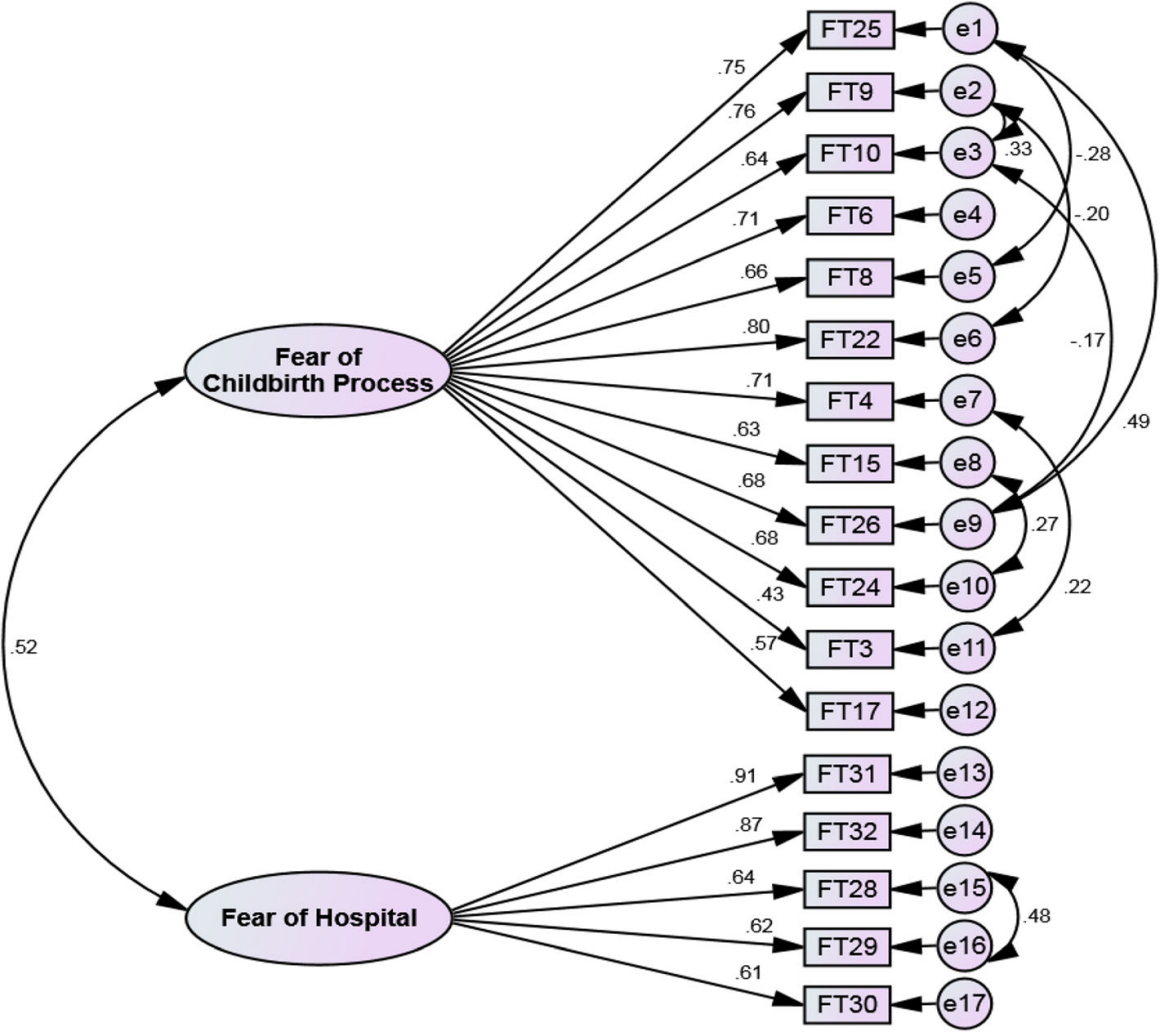
FFCS construct: modified model of first-order confirmation factor analysis

The extracted factor structure was evaluated using CFA and the data obtained from 233 fathers. In the first-order CFA, after modifying the model and drawing the correlation between the measurement errors e_1_ and e_5_, e_1_ and e_9_, e_2_ and e_3_, e_2_ and e_6_, e_3_ and e_9_, e_7_ and e_11_, e_8_ and e_10_, and e_15_ and e_16_, the Chi-squared test for goodness-of-fit was obtained as the first fitting index (χ2 [df = 110, *N* = 233] = 287.67, *p* < 0.001). To evaluate the fitting of the model, other indices were evaluated (IFI = 0.919, CFI = 0.918, AGFI = 0.824, PNFI = 0.707, PCFI = 0.742, RMSEA = 0.083, CMIN/DF = 2.615), which perfectly confirmed the final model (Table 2, Fig. 2). At the end of this stage, the items of FFCS reached to 17.

**Table 2:** Fit indices of the first- and second-order confirmatory factor analysis of the FFCS

CFA Index	IFI	CFI	AGFI	PNFI	PCFI	RMSEA	CMIN/DF	***P***-Value	df	Χ^**2**^
First-order after construct modification	0.919	0.918	0.824	0.707	0.742	0.083	2.615	< 0.001	110	287.670
Second-order after construct modification	0.925	0.924	0.834	0.706	0.740	0.081	2.507	< 0.001	109	273.210

*CFA* Confirmatory Factor Analysis, *CMIN/DF* Chi-square/degree-of-freedom ratio, *RMSEA* Root Mean Square Error of Approximation, *PCFI* Parsimonious Comparative Fit Index, *PNFI* Parsimonious Normed Fit Index, *AGFI* Adjusted Goodness-of-Fit Index, *IFI* Incremental Fit Index, *CFI* Comparative Fit Index. Fit indices: PNFI, PCFI, AGFI (>.5), CFI, IFI (>.9), RMSEA (< 0.08), CMIN/DF (< 3 good, < 5 acceptable)

**Fig. 3:**
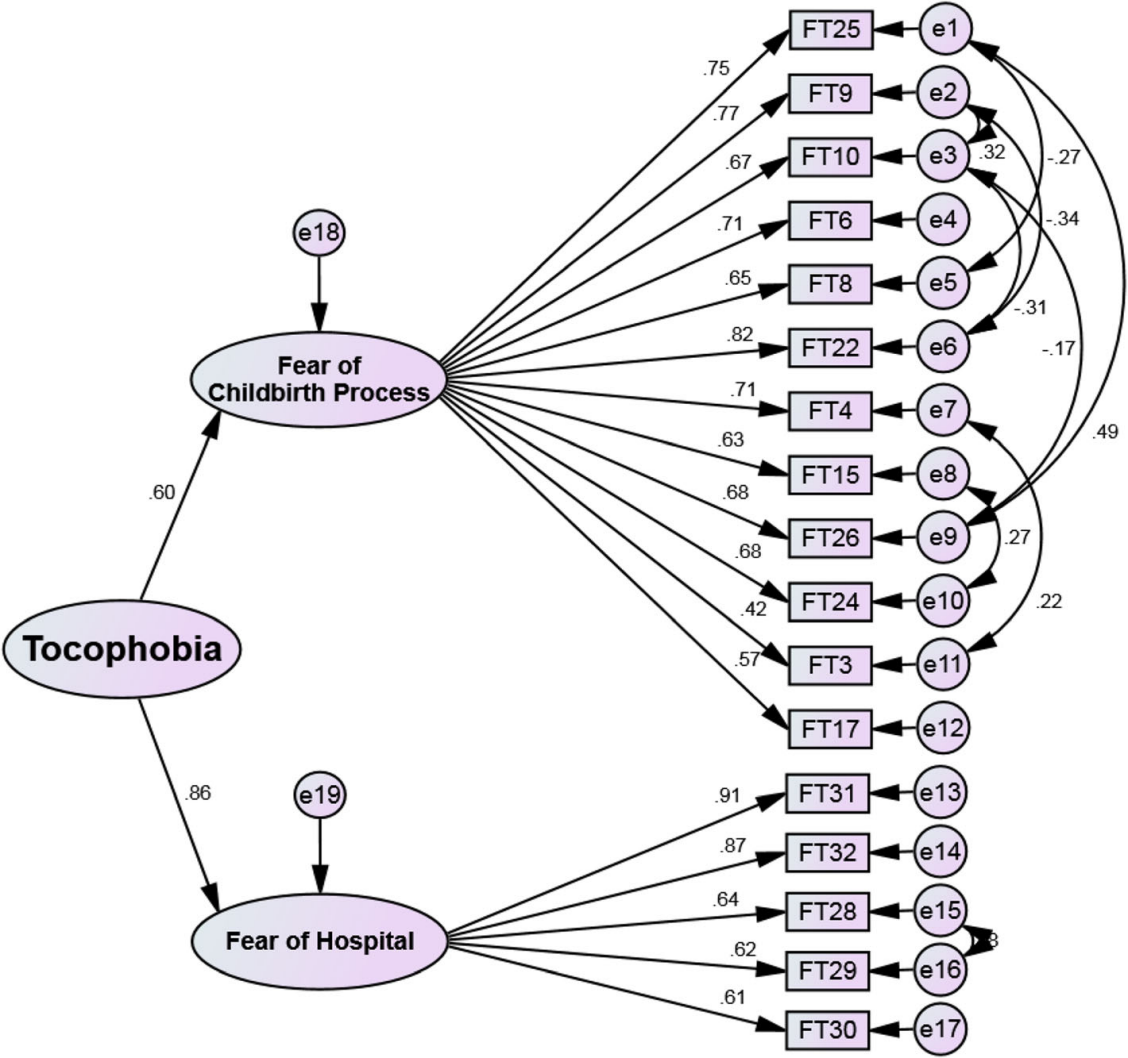
FFCS construct: modified model of second-order confirmation factor analysis

After the first-order CFA, a separate assessment of the factors of the fear of the childbirth in fathers and the correlation between its constructs was performed. The second CFA was conducted to confirm the general concept of “tocophobia”. Figure 3 shows the structural model and the second order CFA of the FFCS with the standardized factor loading coefficients. The amount of factor loading obtained for FFCS was more than 0.5 for all the items, being significant at *p* < 0.001. Internal consistencies of all the factors were greater than 0.70, which confirmed the acceptable internal consistency of the factors (Table 3).

**Table 3:** Reliability indices of FFCS

Index	Alpha (95% CI)	AIC	Omega
Factor			
Fear of Childbirth Process	0.908 (0.889–0.924)	0.449	0.909
Fear of Hospital	0.861 (0.830–0.887)	0.554	0.865

*Alpha* Cronbach’s alpha, *CI* Confidence Interval, *AIC* Average Inter-Item Correlation, *Omega* McDonald’s omega coefficient

### Scoring

The 17 items of the FFCS were scored on a five-point Likert scale (I don’t agree at all _(1)_, I don’t agree _(2)_, I don’t have an opinion _(3)_, I agree _(4)_, I completely agree _(5)_). Therefore, the total score of the scale ranged from 17 to 85 (17–35 low, 36–54 moderate, 55 ≤ high). In this way, the more score indicates the more fear of child birth in the expectant fathers.

## Discussion

The purpose of this study was to design and evaluate the psychometric properties of FFCS. The final FFCS, which showed desirable validity and reliability, included 17 items and two factors consisting of fear of childbirth process (12 items) and fear of hospital (5 items), which explained 50.82% of the total variance. In reliability evaluation, the FFCS showed acceptable internal consistency. The reliability of the total FFCS was established with a Cronbach’s alpha of 0.84, and also the omega was excellent and acceptable.

In this study, the most common indicators of model fitness were evaluated, and all the factor loadings above 0.5 were indicative of a minimum acceptable factor loading. Therefore, based on confirmatory factor analysis, all the fitness indicators had a suitable standard level and the model fitness was appropriate.

The first-order CFA showed that a latent layer was existent, so the secondary-order CFA was used and confirmed the FFCS with two subscales and 17 items. The two subscales represent a more general concept called tocophobia. Tocophobia is comprised of the words “tocos” (a Greek word meaning childbirth) and “phobia” [30]. Tocophobia is considered by its proponents to be a “non-logical fear of childbirth” [31]. The word “tocophobia” as a medical condition was first used by Hofberg and Brockington in 2000 [32]. Tocophobia is divided into two types. Primary tocophobia, which is the destructive fear of childbirth in the first pregnancy, and secondary tocophobia, which, unlike primary tocophobia, is related to the experience of traumatic childbirth in the past [33]. There is little research on men’s experience of tocophobia. Published evidence from India showed that most (78.40%) first-time expectant fathers suffered from tocophobia to various degrees primarily due to concern for the health and life of their partner and child, labor and delivery process, professional competency, hospital staff behaviors, insufficient medical treatment, fear of not being treated with respect and dignity, fear of partners’ and own capabilities, fear of exclusion from decision making, financial matters, and fear of parenting role [34].

The first subscale identified in the exploratory factor analysis was fear of childbirth process. This subscale explained a higher amount of variance than the other subscales. Among the labor-associated fears reported by fathers were seeing the spouse in pain and agony [35], harm to fetus during delivery, being in an unfamiliar situation [36], episiotomy, risk of maternal complications and death associated with cesarean section [6, 37], irreversible rupture [5], prolonged labor [38, 39], and concern for the child’s welfare [40]. Fathers also expressed distress regarding their ability to provide appropriate support to their spouse during labor and childbirth and to react properly to labor-related events [7]. In line with this finding, other research examining the fear of childbirth in fathers has shown that the major fears were related to the health and life of the baby and spouse and the labor and delivery process [5].

Fear of hospital was the second subscale of the FFCS. Hospital may be a very distressing environment for many individuals and this may promote fear in fathers [6]. These findings suggest that some of the risk factors for fear of childbirth were associated with the health care system [5]. In fact, health care providers have been identified as both a cause of fear and a key factor in reducing the fear of childbirth.

A few numbers of instruments have been used to investigate the fear of childbirth in fathers. Among them, the data from this paper indicates that the FFCS may be a better instrument for measuring fear of childbirth in fathers. Although the validity and reliability of Ringler’s questionnaire were confirmed, this questionnaire includes 52 items, which is arguably too time-consuming for fathers to fill out [16]. The W-DEQ is another instrument used for the same purpose, which has two versions for assessing childbirth fear during pregnancy (version A) and after childbirth (version B) [17, 41]. The scales were designed to measure different dimensions of fear of childbirth, though it was ideated as a one-dimensional instrument. Wijma et al. estimated the reliability of the questionnaire by split half and Cronbach’s alpha to be 0.89 and 0.93, respectively [17]. Among the most recent questionnaires on fear of childbirth is the FOBS, which comes in two forms, a single item and a new version with two items [19, 20]. In the new version, the two items measuring fear and worry were strongly correlated (*r* = 0.83). The inclusion of two items allows an estimate of the scale’s reliability (using Cronbach’s alpha), something that is not possible for single-item ratings [19, 42]. In the new version, the Cronbach’s alpha value was 0.91, indicating that the scale has very high internal consistency. Mann–Whitney U test revealed no statistically significant difference between the FOBS scores obtained from the single-item (median = 38, mean = 41.00, SD = 21) and the two items (median = 37, mean = 38.20, SD = 24.10) versions [19]. However, despite the use of these instruments among fathers, there is no accurate data showing that the cut-off score set for the statistical population of pregnant women is also indicative of fear in men [12].

## Implications

According to a statement from the International Conference on Population and Development (ICPD) on men’s participation and responsibility, gender justice should be targeted at all levels of life, including family and social life, and men should be encouraged and empowered to take responsibility for their reproductive and sexual behaviors and their family and social roles. Therefore, today, the role and participation of fathers is emphasized in various aspects of reproductive health, including the childbirth process [43].

On the other hand, the rate of cesarean delivery is ever increasing, and one of the reasons is the growing tendency towards elective cesarean. Studies show that the fear of childbirth in fathers has affected the spike in the rate of cesarean delivery [13]. However, very few studies addressing the fear of childbirth in fathers have been conducted [15, 18]. One of the main reasons for limited studies in this field is the lack of appropriate instruments for use among fathers. Therefore, introducing this instrument may pave the way for further studies on this issue. A valid and reliable scale could be a good starting point for practitioners to engage with fathers. Having a formal scale that could identify areas of concern would enable health care practitioners to address those areas with individual fathers and also systematically within their service.

## Strengths and limitations

The available scales on fear of childbirth are tools targeting the pregnant mothers. The greatest strength of this study is that it developed a specific tool for assessing fear of childbirth in expectant fathers. The other strength of this instrument lies in its development based on empirical data and the existing literature and its construct validity assessment via both exploratory and confirmatory factor analyses. One of our limitations is that this tool was developed in an eastern culture, where fathers may be reluctant to express fear and it was difficult for them to talk about their fears. As a multitude of factors can contribute to the fear of childbirth, psychometric evaluation of this instrument is recommended in different cultural and clinical contexts. Furthermore, the use of a newly designed questionnaire need further psychometric testing at a national level to ensure the robustness of the measure.

## Conclusions

FFCS (Supplementary file 1) is a simple report instrument with proper validity and reliability for the assessment of fear of childbirth in fathers. It can be easily implemented by researchers, midwives, obstetricians and health care providers. This tool allows for designing interventions and studies that may result in turning labor and delivery into a positive experience for fathers in future.

## Supplementary Information

**Additional file 1.**

## Data Availability

The datasets used and analyzed during the current study are available from the corresponding author on reasonable request.

## Abbreviations

FFCS: Father’s Fear of Childbirth
W-DEQ: Wijma Delivery Expectancy/Experience Questionnaire
AIC: Average Inter-Item Correlation
CVR: Content validity ratio
CVI: Content validity index
I-CVI: Item CVI
S-CVI/Ave: Average scale-level CVI
EFA: Exploratory Factor Analysis
CFA: Confirmatory Factor Analysis
IFI: Incremental Fit Index
CFI: Comparative Fit Index
AGFI: Adjusted Goodness of Fit Index
PNFI: Parsimony Normed Fit Index
PCFI: Parsimony Comparative Fit Index
RMSEA: Root Mean Score Error of Approximation
CMIN/DF: Minimum discrepancy function divided by Degrees of Freedom
FOBS: Fear of Birth Scale; SD: Standard Deviation
ICPD: International Conference on Population and Development
