# Correction to: Design and psychometric evaluation of the fathers’ fear of childbirth scale: a mixed method study

**DOI:** 10.1186/s12884-021-03805-6

**Published:** 2021-04-22

**Authors:** Seyedeh Fatemeh Ghaffari, Hamid Sharif Nia, Forouzan Elyasi, Zohreh Shahhosseini, Zohre Mohammadpoorsaravimozafar

**Affiliations:** 1 Mazandaran University of Medical Sciences, Sari, Iran; 2 Amol Faculty of Nursing, Mazandaran University of Medical Sciences, Sari, Iran; 3 Psychiatry and Behavioral Sciences Research Center, Sexual and Reproductive Health Research Center, Addiction Institute, Mazandaran University of Medical Sciences, Sari, Iran; 4 Sexual and Reproductive Health Research Center, Mazandaran University of Medical Sciences, Sari, Iran

**Correction to: BMC Pregnancy Childbirth 21, 222 (2021)**

**https://doi.org/10.1186/s12884-021-03696-7**

Following publication of the original article [1], the authors reported that the correct second author’s name is as below:

First name: Hamid

Family: Sharif Nia

The original article [1] has been updated.

## References

[1] Ghaffari SF, Sharifnia SH, Elyasi F (2021). Design and psychometric evaluation of the fathers’ fear of childbirth scale: a mixed method study. BMC Pregnancy Childbirth.

