# Supplementary material for: Design and psychometric evaluation of the fathers’ fear of childbirth scale: a mixed method study
Source: BMC Pregnancy Childbirth. 2021 Mar 20;21:222. doi: 10.1186/s12884-021-03696-7 (PMC7981919; doi:10.1186/s12884-021-03696-7)
Supplement: Supplementary file 1 — Additional file 1. [file 12884_2021_3696_MOESM1_ESM.docx]

Hello dear father

Respectfully, the expressions in the following questionnaire were extracted to assess the fear of childbirth in fathers. Please read each phrase first and then, based on your current perceptions of childbirth and its stages, mark the answer that best matches your imagination with the * sign.

| Row | The desired indicator | I don't agree at all | I don't agree | No idea | I agree | I completely agree |
| --- | --- | --- | --- | --- | --- | --- |
| 1 | I worry about the quality of sex with my spouse after childbirth. |  |  |  |  |  |
| 2 | As the time of childbirth approaches, my worries increase. |  |  |  |  |  |
| 3 | I am afraid that dangerous medical interventions will be needed during childbirth. |  |  |  |  |  |
| 4 | During my spouse's childbirth, I will feel helpless. |  |  |  |  |  |
| 5 | During my spouse's childbirth, I will feel fear. |  |  |  |  |  |
| 6 | During my spouse's childbirth, I will feel restless. |  |  |  |  |  |
| 7 | Because of my spouse's fear of childbirth, I feel fear. |  |  |  |  |  |
| 8 | I am afraid that I am not capable enough to support my spouse during childbirth. |  |  |  |  |  |
| 9 | I'm afraid that my spouse's childbirth will be risky. |  |  |  |  |  |
| 10 | I will feel fear because of my spouse 's pain. |  |  |  |  |  |
| 11 | I am afraid that my spouse's health will be endangered due to childbirth |  |  |  |  |  |
| 12 | I am afraid that my child's health will be endangered due to childbirth. |  |  |  |  |  |
| 13 | I am afraid that the hospital staff will not take enough care of my spouse. |  |  |  |  |  |
| 14 | I'm afraid the hospital staff won't treat me and my spouse respectfully. |  |  |  |  |  |
| 15 | I am afraid that my child will be hospitalized in the neonatal intensive care unit after birth. |  |  |  |  |  |
| 16 | I am afraid that the hospital staff will not have enough skills to perform a safe childbirth. |  |  |  |  |  |
| 17 | I am afraid that the hospital will not have enough facilities and equipment for a safe childbirth. |  |  |  |  |  |
